# Tropical Almond Tree (*Terminalia catappa* L.): A Comprehensive Review of the Phytochemical Composition, Bioactivities and Economic Potential

**DOI:** 10.3390/ph19010099

**Published:** 2026-01-05

**Authors:** Oscar Zannou, Nour M. H. Awad, Vénérande Y. Ballogou, Sarhan Mohammed, Yann Emmanuel Miassi, Marcel Houngbédji, Kossivi Fabrice Dossa, Adam Abdoulaye, Mohamed Ghellam, Yénoukounmè E. Kpoclou, Midimahu V. Aïssi, Gulden Goksen, Ilkay Koca, Reza Tahergorabi

**Affiliations:** 1 Department of Nutrition, Food Sciences and Technology, Faculty of Agricultural Sciences, University of Abomey-Calavi, Cotonou 01 BP 526, Benin; 2 Department of Food Technology, Vocational School of Technical Sciences at Mersin Tarsus Organized Industrial Zone, Tarsus University, 33100 Mersin, Türkiye; awadnur5@gmail.com (N.M.H.A.); guldengoksen@tarsus.edu.tr (G.G.); 3 Unité de Recherche en Génie Enzymatique et Alimentaire, Laboratoire d’Etude et de Recherche en Chimie Appliquée, Ecole Polytechnique d’Abomey-Calavi, University of Abomey-Calavi, Cotonou 01 BP 2009, Benin; ballogouvnrande@yahoo.fr; 4 Department of Food Engineering, Faculty of Engineering, Ondokuz Mayıs University, 55139 Samsun, Türkiye; sar1721155@gmail.com (S.M.); itosun@omu.edu.tr (I.K.); 5 Faculty of Forestry, Geography and Geomatics, Laval University, Quebec, QC G1V 0A6, Canada; yannmanu006@gmail.com (Y.E.M.); fabdossa@gmail.com (K.F.D.); 6 Laboratory of Food Sciences, University of Abomey-Calavi, Cotonou 03 BP 2819, Benin; houngbedjimarcel@gmail.com; 7 Laboratory of Microbiology and Food Technology, Department of Plant Biology, Faculty of Science and Technology, University of Abomey-Calavi, Cotonou 01 BP 4521, Benin; adamabdoulaye42@yahoo.fr; 8 Food Science Laboratory (LSA), Department of Food Engineering, ISVSA, University of Batna, Batna 05000, Algeria; mohamed.gh2010@gmail.com; 9 Laboratoire de Science et Technologie des Aliments et Bioressources et de Nutrition Humaine, Ecole des Sciences et Techniques de Conservation et de Transformation des Produits Agricoles, Université Nationale d’Agriculture, Sakété BP 114, Benin; euloge.kpoclou@gmail.com (Y.E.K.); vahidaissi@yahoo.fr (M.V.A.); 10 Food and Nutritional Sciences Program, North Carolina A&T State University, Greensboro, NC 27411, USA; rtahergo@ncat.edu

**Keywords:** *Terminalia catappa* L., nutrition, phytochemicals, bioactive compounds, economic potential

## Abstract

Tropical almond tree (*Terminalia catappa* L.), belonging to the Combretaceae family, is an unfurling tree with different edible parts. This review discussed the nutritional content, ethnopharmacological applications, main bioactive components, biological effects and economic potential of *T. catappa*. *T. catappa* shows essential applications in medicine, cosmetics and pharmaceutics. The nutritional values of *T. catappa* are associated with its contents of carbohydrates, minerals, proteins, lipids, vitamins and amino acids. It is used in many ethnopharmacological applications, including a heart stimulator, anti-diarrhoeal, bactericidal, anti-parasitic and anti-stress. *T. catappa* is used to treat angina pectoris, asthma attacks and bronchitis. The main reported biological activities for *T. catappa* were antioxidant, antidiabetic, anti-atherosclerosis, antitumor, antimicrobial, anthelmintic, antimalaria, hepatoprotective, insecticidal, anti-inflammatory and antihyperlipidemic activities. The main bioactive components reported in *T. catappa* encompassed phenolic compounds, alkaloids, diterpenes, fatty acids, galloyl glucose and derivatives, steroids and coumarins. *T. catappa* shows great economic opportunities which need to be expanded and diversified, taking into account its sustainability.

## 1. Introduction

Africa has a rich and varied flora that has always been an inexhaustible source of culinary discoveries. Among this diversity, the tropical almond tree (*Terminalia catappa*), emblematic of arid lands, stands out. Belonging to the Combretaceae family, *T. catappa* is a fruit tree. It can reach a height of around twenty meters. *T. catappa* is a great and unfurling tree that normally emerges in drained and well-aerated sandy soils [1]. It propagates from the seeds and grows faster with minimum maintenance; however, the breeding and improvement lack documentation [2]. It has tiered branches with large and smooth leaves forming a bunch. The leaves are deciduous, simple, slightly oval, with a short and thick stalk. They turn from yellow to red before dropping. *T. catappa* loses almost all its leaves twice a year, in January–February and July–August. The fruits have an ellipsoidal form, a thin epicarp and fibrous mesocarp [3]. They turn from green to yellow or red or purple when they ripen. The smooth outer skin of the fruits covers a layer of cork-like fibers surrounding the flesh. *T. catappa* has a kernel containing a very pleasant almond. *T. catappa* is usually used for ornaments, shading and the production of biofuels [4]. It is also a good source for the production of briquettes, with excellent physical and mechanical properties [5].

In African countries and Asia, *T. catappa* is used for medicinal purposes [6,7]. In recent decades, an increasing interest has been shown to *T. catappa* due to its high content of phytochemicals and nutrients [8]. *T. catappa* displayed beneficial health properties, including antifungal, antioxidant, anti-inflammatory, antibacterial and nephroprotective [6,7,9,10,11]. Many phytochemicals, including phenolic acids, flavonoids, fatty acids and tannins, have been reported in various parts of *T. catappa* [12,13]. The kernel contains good amounts of lipids, proteins and minerals (magnesium, potassium, calcium and zinc) [14,15].

This review attempts to comprehensively analyze the phytochemical and nutrition diversities, ethnopharmacological basis, biological activities and economic potential of *T. catappa* L. This comprehensive review compiled all the relevant data in the period between 2000 and 2025, which dealt with the different parts of *T. catappa* L. and their phytochemical constituents, nutritional values and biological activities.

## 2. Data Collection Methodology

An online thorough literature search was conducted by combining the relevant keywords, including tropical almond tree, *Terminalia catappa*, traditional uses, ethnopharmacology, nutrient contents, bioactive compounds, biological activity and economic potentials. The search was carried out by using “AND”/“OR”. The search was extended from 2000 to 2025 by using PubMed, ScienceDirect, Web of Science, Google Scholar and Reaxys. The final search was performed on 23 December 2025. References were arranged using the Mendeley software (Mendeley Desktop1.19.8).

## 3. Traditional Uses and Ethnopharmacology

**Figure 1:**
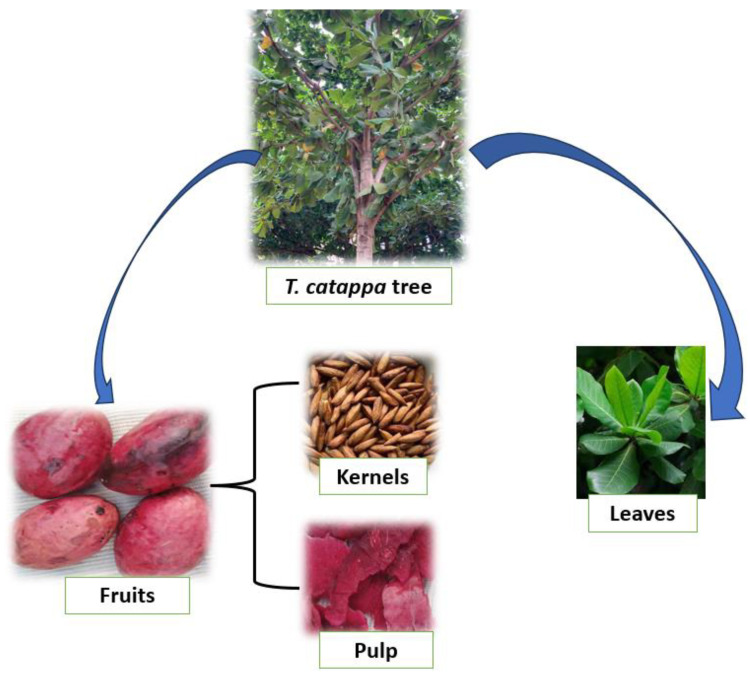
*T. catappa* and by-products.

*T. catappa* (Figure 1) is a characteristic tree of the savannahs of West and Central Africa which has become naturalized in many tropical regions including Benin, Ghana, Nigeria, Brazil, Côte d’Ivoire, India and Thailand. *T. catappa* is widespread in open deciduous forests and scrubby grassy savannahs. It is often found near rivers on poorly drained clay soils. *T. catappa* can be propagated by its seed and by cuttings. Although its thick, corky bark makes it fairly fire-resistant, *T. catappa* is threatened locally by overexploitation. *T. catappa* nuts contain an edible kernel with a delicate flavor. They are eaten at the foot of the tree, after breaking the shell between two stones. They can also be sold dried in urban markets. These kernels can also produce drinks and roasted kernels coated in caramel [16]. Moreover, fresh or dried, the kernel can be eaten directly [17]. These kernels can also be used to produce drinks and roasted kernels coated in caramel [16].

Recently, the WHO emphasized the importance of traditional and complementary medicine for the health of the population, as well as the increasing demand for these medical practices due to the elevated number of chronic diseases [18]. Different parts of *T. catappa* are traditionally used for the treatment of various illnesses [19,20]. *T. catappa* is widely used in traditional medicine across Africa, Asia and Latin America. Its ethnopharmacological importance is linked to the extensive use of its leaves, bark, fruits and seeds to treat various ailments. The leaf juice or ointments are applied for the relief of inflammatory and skin diseases—for leprosy, scabies, dermatitis, cutaneous infections, wounds and ulcers [21,22,23]. The bark and leaves are used for the treatment of gastrointestinal disorders such as dysentery, diarrhea, colitis, dyspepsia and intestinal parasites [22,24,25]. In folk medicine, the leaves or barks of Terminalia species are used for the treatment of skin diseases, hepatitis and other liver problems [23,24,26]. Furthermore, the bark and leaves of *T. catappa* are traditionally used as a heart stimulator, anti-diarrhoeal, bactericidal, anti-parasitic and anti-stress. *T. catappa* is used to treat angina pectoris, asthma attacks and bronchitis. The fruits or leaves are used for the treatment of metabolic and cardiovascular conditions, including diabetes, hypertension, hypercholesterolemia and cardiovascular disorders [9,12,22,27]. Moreover, in Ayurveda, *T. catappa* is used to cure cough, asthma, fever, rheumatism, headache, general tonic and “restoring the power of senses” [21,22,24,28].

Abiodun et al. [10] stated that *T. catappa* is frequently used in tropical regions as having anti-inflammatory, anti-diarrhoeal and antioxidant properties. In West African regions, many parts of the plant are commonly used in traditional medicine to treat a wide range of illnesses, including malaria, hepatitis, venereal diseases and conjunctivitis. The roots are used to treat depression, coughs, syphilis, urinary infections, female sterility, tuberculosis, snake bites and skin diseases, fever and high blood pressure. In India, *T. catappa* leaves are used for the treatment of many illnesses including scabies, leprosy wounds and other skin diseases. They are also traditionally used for the treatment of diarrhea and fever in many Asian regions [29]. Silva et al. [30] mentioned *T. catappa* as a medicinal plant from the Caribbean to treat gastritis.

## 4. Nutrient Contents of *T. catappa*

**Figure 2:**
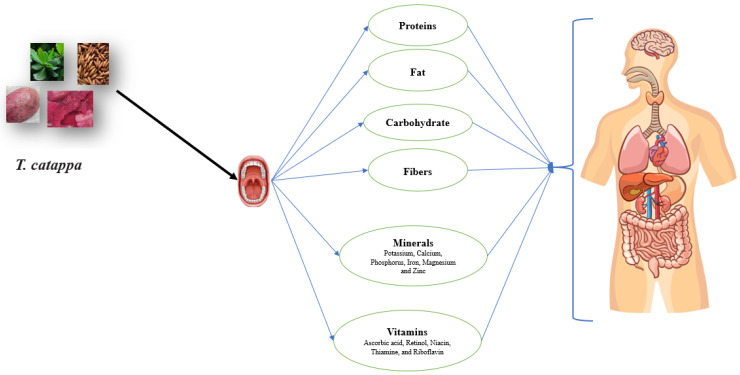
Nutritional properties *T. catappa* by-products.

The nutritional content is one of the determining criteria to be taken into account to assess the fruit quality and its application in industry. The nutritional content of fruit is dependent on its intrinsic characteristics and the environmental conditions in which the tree grows. The nutritional content of *T. catappa* differed greatly from each plant organ (Table 1; Figure 2). The highest moisture content was found in the pulp (16.54%), followed by the leaves (10.6%), kernel (6.23–6.92%) and flower (5%), respectively [14,31,32,33,34]. However, the highest protein content was detected in the kernel, being 21.98–25.95%, followed by the leaves (8.73%), kernel (8.28%) and pulp (2.54%), respectively (Table 1). The carbohydrate content also varied in each plant organ, with the flower displaying the highest value (30.85%), followed by the pulp (11.27%) and kernel (6.88–7.01%), respectively (Table 1).

A study addressed the nutritional content of *T. catappa* seeds from Malaysia [33]. The seeds contained moisture of 6.23%, ash of 3.78%, lipid of 54.68%, protein of 17.66%, total dietary fiber of 9.97%, carbohydrate of 7.68, reducing sugar of 1.36% and starch of 1.22%. The seeds contained considerable amounts of amino acids, including glutamic acid (5.69 ± 1.61 g/100 g), arginine (3.95 g/100 g), aspartic acid (2.15 g/100 g), isoleucine (1.79 g/100 g), glycine acid (1.69 g/100 g), cysteine (1.40 g/100 g), leucine (1.15 g/100 g), phenylalanine (1.14 g/100 g), serine (1.12 g/100 g), threonine (1.04 g/100 g), valine (1.03 g/100 g), alanine (1.02 g/100 g) and proline (1.02 g/100 g) [33].

Dos Santos et al. [32] reported the proximate composition of *T. catappa* almond pulp to be moisture (16.54%), ashes (4.11%), proteins (2.54%), lipids (14.95%), carbohydrate (11.27%), total fibers (31.68%) and total energy value (268.07 Kcal/100 g). Similarly, Jahurul et al. [14] reported the nutritional contents of *T. catappa* kernel as moisture (6.87–6.92%), ash (4.77–4.54%), protein (22.44–21.98%), total fat (54.47–49.65%), carbohydrate (6.88–7.01%) and total fiber (5.13–5.36%). The kernel of *T. catappa* almond from Sri Lanka contained moisture (2.44–2.88%), protein (25.22–25.95%), fat (60.3666.25%), ash (4.03–4.30%) and total carbohydrate (1.62–6.95%) [35]. The mineral composition of the kernel of *T. catappa* was evaluated and it was revealed that the major minerals are potassium (6861.41–7575 mg/kg), the most abundant element, followed by calcium (2294–2687.52 mg/kg) and magnesium (2273.92–2529.37 mg/kg) [35].

Dos Santos et al. [32] reported the proximate composition of *T. catappa* almond pulp to be moisture (16.54%), ashes (4.11%), proteins (2.54%), lipids (14.95%), carbohydrate (11.27%), total fibers (31.68%) and total energy value (268.07 Kcal/100 g). Similarly, Jahurul et al. [14] reported the nutritional contents of *T. catappa* kernel as moisture (6.87–6.92%), ash (4.77–4.54%), protein (22.44–21.98%), total fat (54.47–49.65%), carbohydrate (6.88–7.01%) and total fiber (5.13–5.36%). The kernel of *T. catappa* almond from Sri Lanka contained moisture (2.44–2.88%), protein (25.22–25.95%), fat (60.3666.25%), ash (4.03–4.30%) and total carbohydrate (1.62–6.95%) [35]. The mineral composition of the kernel of *T. catappa* was evaluated and it was revealed that the major minerals are potassium (6861.41–7575 mg/kg), the most abundant element, followed by calcium (2294–2687.52 mg/kg) and magnesium (2273.92–2529.37 mg/kg) [35].

One study addressed the vitamin and mineral contents of the endocarp of *T. catappa* fruit from Nigeria [34]. The flour of the fruit endocarp of *T. catappa* contained elevated content of moisture (5.00%), ash (11.03%), crude fiber (36.33%), crude fat (8.53%), crude protein (8.28%) and carbohydrate (30.85%). Potassium and calcium were found at higher levels compared to magnesium, phosphorus and sodium, while ascorbic acid was found as the most abundant vitamin, followed by retinol, niacin, thiamine and riboflavin [34]. A study was carried out on the nutritional composition of the leaves of *T. catappa* from Ghana [31]. They reported dry matter of 89.4%, organic matter of 92.4%, ash of 7.63%, crude protein of 8.73% and crude fiber of 18.3%. The leaves of *T. catappa* contained nitrate (1710 mg/kg), nitrite (5912.5 mg/kg), sulfate (1612.5 mg/kg), phosphorous (469.77 mg/kg), copper (41.5 mg/kg), magnesium (825 mg/kg), nickel (729.5 mg/kg), iron (5 mg/kg) and ammonium (9.05 mg/kg) [25]. In short, the nutritional values of *T. catappa* vary greatly depending on the plant organ considered, as well as the origin of the raw material. For instance, the studied data revealed that the kernel, pulp and leaves of *T. catappa* are high-energy and nutrient-rich foods suitable for oil extraction and formulation of functional foods.

**Table 1:** Nutrient composition of leaves, pulp, kernel, and flower of *T. catappa*.

Plant Part	Nutrient Content	References
**Leaves**		
Moisture	10.6%	[31]
Crude protein	8.73%	[31]
Crude fibers	18.3%	[31]
Ash	7.63%	[31]
Phosphorous (P)	469.77 mg/kg FW	[25]
Calcium (Ca)	49,656 mg/kg DW	[36]
Magnesium (Mg)	825 mg/kg FW	[25]
Total phenolic	612.26 g T.A/kg DM	[37]
Total tannins	586.19 g T.A/kg DM	[37]
**Pulp**		
Moisture	16.54%	[3]
Ash	4.11%	[3]
Protein	2.54%	[3]
Lipids	14.95%	[3]
Carbohydrate	11.27%	[3]
Starch	19.57%	[3]
Total fibers (%)	31.68%	[3]
Total phenolic	361.18 g T.A/kg DM	[37]
Total tannins	298.93 g T.A/kg DM	[37]
**Kernel**		
Moisture	6.23–6.92%	[14,33]
Protein	21.98–25.95%	[14,33,35]
Starch	1.22%	[33]
Total fiber	5.13–5.36%	[14]
Carbohydrate	6.88–7.01%	[14]
Oil content	49.45–60.45%	[14,38]
Saturated fatty acids	36.16 to 41.33%	[14,35,38]
Unsaturated fatty acid	59.17% to 63.2%	[14,35,38]
Ash	4.54 to 4.77%	[14]
Potassium (K)	6861.41–7575 mg/kg	[35]
Calcium (Ca)	2294–2687.52 mg/kg	[35]
Magnesium (Mg)	2273.92–2529.37 mg/kg	[35]
Zinc (Zn)	32.72–42.57 mg/kg	[35]
Copper (Cu)	22.09–22.31 mg/kg	[35]
Manganese (Mn)	13.91–20.95 mg/kg	[35]
Barium (Ba)	10.28–10.97 mg/kg	[35]
Strontium (Sr)	4.30–7.26 mg/kg	[35]
Boron (B)	2.95–3.72 mg/kg	[35]
Cobalt (Co)	0.34–1.08 mg/kg	[35]
Silver	0.02–0.04 mg/kg	[35]
Total phenolic	39.10–55.97 mg GAE/g	[14]
Total tannins	282.84 g T.A/kg DM	[37]
Aspartic acid	2.15 g/100 g	[33]
Glutamic acid	5.69 g/100 g	[33]
Serine	1.12 g/100 g	[33]
Glycine acid	1.69 g/100 g	[33]
Histidine	0.52 g/100 g	[33]
Arginine	3.95 g/100 g	[33]
Threonine	1.04 g/100 g	[33]
Alanine	1.02 g/100 g	[33]
Proline	1.02 g/100 g	[33]
Tyrosine	0.76 g/100 g	[33]
Valine	1.03 g/100 g	[33]
Methionine	0.26 g/100 g	[33]
Cysteine	1.40 g/100 g	[33]
Isoleucine	1.79 g/100 g	[33]
Leucine	1.15 g/100 g	[33]
Phenylalanine	1.14 g/100 g	[33]
Lysine	0.42 g/100 g	
**Flower**		
Moisture	5%	[34]
Crude protein	8.28%	[34]
Crude fibers	36.33%	[34]
Oil content	8.53%	[34]
Carbohydrate	30.85%	[34]
Ascorbic acid	14.58 mg/100 g FW	[34]
Ash	11%	[34]
Phosphorous (P)	41.87 mg/100 g	[34]
Potassium (K)	326.32 mg/100 g	[34]
Calcium (Ca)	220.19 mg/100 g	[34]
Magnesium (Mg)	43.66 mg/100 g	[34]

T.A: Tannic acid, GAE: Gallic acid equivalent, DW: Dry weight, FW: Fresh weight.

## 5. Phytochemical Composition

### 5.1. Phenolics

**Figure 3:**
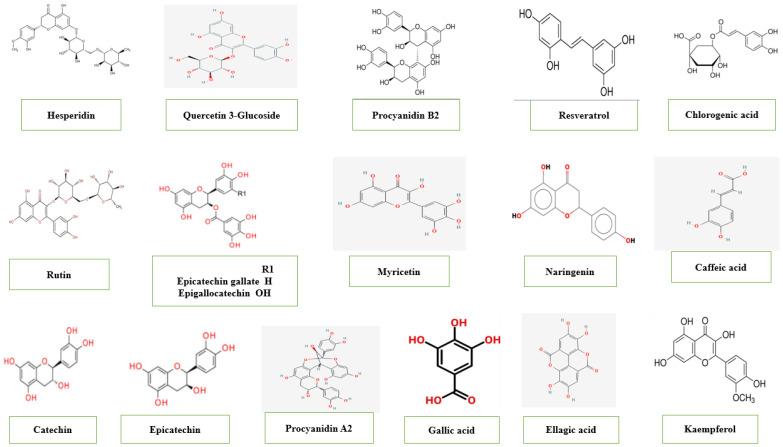
Major phenolic compounds of *T. catappa*.

*T. catappa* is a significant source of bioactive components (Figure 3) [14,39]. However, the bioactive content of *T. catappa* trees is subject to several considerations. The phenolic content of the kernel oil of Kota Kinabalu and Keningau cultivars was reported to be 55.97 mg GAE/g, while a total phenolic content of 39.10 mg GAE/g was found for the Keningau cultivar [14]. Hence, the higher phenolic content in the oil coincided with low rainfall in the Kota Kinabalu cultivar. In contrast, high rainfall regions may cause high oil content in *T. catappa* kernels [39]. In *T. catappa* leaves, a significant amount of alkaloids, tannins, steroids, cardiac glycosides, flavonoids, saponins and coumarins have been identified [40,41]. Although terpenoids were absent in Mwangi et al. [41], they were determined as a sesquiterpene (C_15_H_18_O_3_) in a previous study [42]. Uchida et al. [43] recommended the use of the water–ethanol mixtures as an effective solvent to increase the extraction yields from *T catappa* fruit pulp. It showed high concentrations of phenolic compounds were 2.98 mg/mL, flavonoids were 1.644 mg/g, anthocyanins were 0.505 mg/g and flavonols were 0.123 mg/g. The quantitative analysis of *T. catappa* leaves has significant phenolic contents in the ethanolic extract. The results of the ethanolic extract showed that the highest phenol and flavonoid content (21.56 mg GAE/g and 1.31 mg QE/g dry matter) was compared to ethanolic–water and aqueous extracts. [44] Total phenols (GAE) and flavonoids (QE) contents from the ethanol extract of leaves were found to be 354.02 and 51.67 mg/g extract. [45] Chromatograms of the hot aqueous extract (HAE) and room temperature of *T. catappa* leaves showed similar chemical compounds; however, higher levels of compounds were in HAE. Chemical compounds are identified as gallic acid, ellagic acid and punicalagin. [46] Moderate phenolic contents were found in both fermented and unfermented *T. catappa* leaves from Ondo State, Nigeria. [47] In both ethanolic extracts, saponin and p-coumaric acid are the most abundant compounds at 1009.24 mg/g–436.95 mg/g and 1666.03–552.02 mg/g, respectively. Other compounds include quercetin (53.50–217.81 mg/g) and naringenin (61.08–56.14 mg/g). Caffeic acid (2.05 mg/g) and tannic acid (5.24 mg/g) were the least concentrated in fermented and unfermented extracts, respectively. Studies showed higher phenolic contents in the unfermented *T. catappa* leaves; however, additional bioactive compounds were observed in the fermented type, including salicylic acid (14.59 mg/g) and apigenin (72.27 mg/g). In the unfermented and fermented *T. catappa* seeds, the total phenolic content, flavonoid and tannin were quantified to be 3.214–3.003 mg (GAE)/g, 0.756–0.696 mg (RE)/g, and 0.103–0.087 mg (TAE)/g have been reported, respectively [48]. *T. catappa* leaves are tannin-rich components, especially ellagitannins (hydrolyzable tannins), which are linked to the antitumor [26,49]. Moreover, phytochemicals present in *T. catappa*, such as tannins, flavonoids, gallotannins, cyanidins and ellagitannins, showed anti-HIV activity [50]. The chemical analysis of *T. catappa* leaves (São Luís, Brazil) detected the presence of hydrolyzable tannins (punicalin and punicaligin), gallic acid and C-flavonoid glycosides [26]. Moreover, Chen et al. [49] reported that *T. catappa* leaves water extract contains 21% tannin. Tannins and flavonoids, constituents of the aqueous leaf extract of *T. catappa*, were 0.32 ± 0.00 and 1.68%, respectively [51]. The percentage of tannins (0.11%) is found in the gum of *T. catappa* (Almendron, in Venezuela) [52]. Raw *T. catappa* nut demonstrated tannins with 27.13 μg/g, which was better than roasted nuts [8]. In *T. catappa* fruit (Nigeria), the pericarp was found rich with phenol (149.33 GAE/100 g), while the seed was saponin (84.15 mg/100 g) [53]. Study on *T. catappa* bark (Karnataka, India) highlights its potential as a source of bioactive compounds [54]. Alcoholic extract (TCE) and water extract (TCW) were prepared. The TPC of both extracts was found to be 287 and 175 mg/g, respectively. Thus, the TFC was calculated as 10.2 and 61.7 mg QE/kg for TCE and TCW, respectively. *T. catappa* can well compete with *P. amygdalus* in terms of bioactive compounds. *P. amygdalus* (sweet almond) was found to be higher in phytochemicals than *T. catappa* (tropical almond). In sweet almonds, tannin, phenols, flavonoids and saponins were 748.49, 1781.50, 456.38 and 158.70 μg/g, respectively. In tropical almonds they were 388.95, 410.83, 73.28 and 86.32 μg/g, respectively [55]. The differences are attributed to several factors, such as drying, climatic conditions and other processing methods.

### 5.2. Alkaloids

*T. catappa* can well compete with *P. amygdalus* in terms of phytochemicals such as alkaloids. In both tropical almonds and sweet almonds, no significant differences were found (*p* > 0.05). Alkaloids were found to be 210.65 μg/g and 240.11 μg/g in tropical almond and sweet almond, respectively [55]. In the aqueous leaf extract of *T. catappa*, the alkaloid constituent was 0.37% [51]. Nuts of both the yellow and red varieties of *T. catappa* were investigated. Results indicated that alkaloids are present in the red variety, such as anthraquinones, while terpenoids and steroids are in the yellow variety [56]. *T. catappa* nuts presented an abundance of alkaloids such as ribalinidine, quinine and sparteine, with anti-malaria and antioxidant potential [8]. Raw *T. catappa* nut demonstrated quinine, ribalinidine and spartein with 7.18, 45.57 and 7.33 μg/g, which was better than roasted nuts. In *T. catappa* fruit (Nigeria), alkaloids were present in the pericarp (34.42 mg/100 g), but not found in the seed. [16] Moreover, glycoside and phlobatannin were found to be absent in the seed. *T. catappa* was reported to contain alkaloids with pharmacological activities such as antimicrobial activity [41,57]. It is able to interchelate with the DNA of both Gram-negative and Gram-positive bacteria [58]. Alkaloid in unfermented and fermented *T. catappa* seeds was determined to be 24.51% and 20.61%, respectively [48].

### 5.3. Fatty Acids

One more important part of *T. catappa* is its kernel. *T. catappa* kernel has a relatively high oil yield. The kernel oil of *T. catappa* is commonly referred to as *T. catappa* kernel oil (TCKO). TCKO yield ranges from 49 to 65% (Table 2) [59,60]. Compared to other commercial oil sources, TCKO yield is higher than cottonseed and soybean [2]. In addition to seeds containing high levels of oil content (600 g/kg), it has the ideal ratio of fatty acids as recommended by dietary guidelines [61]. Despite this importance, it is still considered underutilized. Therefore, it can be used as one potential alternative to some conventional oils in countries where it is available. Several factors play a role in oil yields, including (1) territorial origin, (2) soil nature, (3) harvest time, (4) extraction processing, (5) the age of the tree and fruit maturity or (6) precipitation rate [39,62]. Although Mbah et al. [63] indicated that the fat of the sample did not change concerning the process and location, Jahurul et al. [14] confirmed that the total oil content of *T. catappa* kernel cultivated from Kota Kinabalu was higher than that of Keningau. Table 2. represents the fatty acid composition of different *T. catappa* kernel oil. The oil extraction yield in *T. catappa* (Sabah, Malaysia) ranges from 49.45 to 54.47%. Moreover, the result showed that free fatty acid values are quantified to be 1.17 and 2.42% [14]. Soxhlet extraction of *T. catappa* kernel oil yielded higher than the maceration methods. The result showed that the major SFAs and MUFAs in *T. catappa* kernel oil are palmitic acid (C16:0) and oleic acid (C18:1). A study by Iha et al. [59] quantified total saturated fatty acid and total unsaturated fatty acid in *T. catappa* kernel to be 34.2% and 64.5%, respectively. In the Brazilian *T. catappa*, castanhola (60%) was found as the predominant fatty acids [64]. Moreover, within unsaturated fatty acids in the *T. catappa* fruit seed oils, oleic and linoleic acids were predominant [65]. The oleic and linoleic acids together account for 53.47% of *T. catappa* seed oil [66]. Cuticular waxes in *T. catappa* leaves include mainly fatty acids [67]. In Malaysian-grown tropical almond nuts, the seed oil content was determined to be 54.68% [33]. Fatty acid composition of Nigerian-grown tropical almonds was 43.71% [55]. GC-MS analysis of n-hexane extract of *T. catappa* nuts from Nigeria revealed the presence of several notable bioactive compounds. The most abundant component identified was cis-vaccenic acid (24.493%). Overall, fat represented 56.71% of the nut composition [68]. Results of *T. catappa* seeds (Malaysia) revealed that palmitic acid and oleic acid were the major saturated and unsaturated fatty acids, respectively [33]. The proximate analysis found that the crude fat content of plant-based milk using local almond nuts was higher than that of cow’s milk [69].

**Table 2:** Fatty acid composition of different sources *T. catappa* kernel oil.

Fatty Acids	Origins						
	Nigeria	Brazil	Malaysia	Nigeria	Malaysia	Brazil	Ivory Coast
Lauric acid	0.94	-		-	-	-	-
Myristic acid	0.54	0.10	0.08	-	0.09	-	0.17
Palmitic acid	36.01	28.30	31.32	34.82	28.98	35.0	36.20
Palmitoleic acid	-	0.90	0.31		0.34		0.41
Stearic acid	6.4	4.90	5.17	6.79	7.23	5.0	4.02
Oleic acid	33.25	30.00	28.62	30.13	39.28	32.0	27.97–32.40
Linoleic acid	22.26	32.80	32.25	23.44	23.01	28.0	24.65–31.67
Linolenic acid	0.59	1.70	0.09	-	0.07		0.55
Saturated fatty acids (%)	43.89	34.20	37.57	43.92	-	-	40.22–42.98
Mono-unsaturated fatty acid (%)	33.25	30.00	29.03	-	-	-	27.97–32.81
Polyunsaturated fatty acid (%)	22.85	34.50	32.34	-	-	-	25.20–32.17
Unsaturated fatty acids (%)	56.10	74.50	61.37	56.08	-	-	-
References	[38]	[59]	[14]	[66]	[33]	[64]	[65]

### 5.4. Volatile Constituents and Essential Oil Compositions

Essential oils are vital ingredients of the tropical almond tree. They are abundantly present in the leaves, barks, seeds and nuts of the *T. catappa*. The investigation of *T. catappa* leaves essential oil via hydrodistillation and GC/MS analysis revealed the presence of volatile components. Ogunmoye et al. [70] detected 40, of which the most abundant were identified as hexahydrofarnesyl acetone (12.34%), 1,3,8-p-menthatriene (9.38%) and 1,2-dimethyl-cyclooctene (7.30%). Another study with the same approach found that (Z)-phytol (41.2%), palmitic acid (11.0%) and (E)-nerolidol (4.7%) were the main identified components [71]. In fruit oil extraction, α-farnesene (21.3%), octadecane (11.7%) and palmitic acid (9.5%) were the major constituents [72]. Hydrocarbons (21.61%) were identified as an abundant class of compounds in *T. catappa* air-dried leaves oil [70]. Twelve volatile constituents in almond methanol leaf extract were detected; the main identified component was 9, 17-Octadecadienal-Z [73]. DL-α-tocopherol (28.67%), phytol (23.30%), squalene (14.83%) and β-sitosterol (13.92%) were identified in the ethanolic leaf extract of *T. catappa* through GC-MS analysis [74]. By employing GC-MS, palmitic acid (33.2%), linoleoyl chloride (29.1%) and pentadecanolide (16.2%) were revealed as the main components in *T. catappa* nuts [75]. A total of 21 volatile compounds in *T. catappa* seed oil were identified. The n-Hexadecanoic acid (17.96%), oleic acid (22.42%) and 2,9-octadecadienoic acid (22.82%) were detected [76]. Twenty phytochemicals could be identified in both raw and roasted tropical almond nuts from Nigeria. Upon roasting, results showed that quinine, ribalinidine, sapogenin, flavan-3-ol and tannin were reduced, whereas catechin seemed enhanced [8]. Ripe fruits of T. catappa in Brazil were screened for volatile constituents using GC-MS profiling. Results showed that the majority were phenols (50%), furan derivatives and cyclic ketones, with a minor amount of alcohols and esters [77].

### 5.5. Triterpenoid

Triterpenoids are organic compounds of 30 carbon atoms, which are formed by the polymerization of six isoprene units, and are widely found in nature in different chemical structures [78]. *T. catappa* L. contains triterpenoids in various structures including ursolic acid and asiatic acid. These compounds have demonstrated hepatoprotective properties through the protection of liver mitochondria and the scavenging action on free radicals [79]. Generally, terpenoid compounds are commonly reported in the leaves, seed and fruit pulp of *T. catappa* at a concentration of up to 0.22–0.31 mg/g in the fruits [80]. Common terpenoid compounds reported in this plant include oleanolic acid, 2-alpha, 3-beta, 23-trihydroxyurs-12-en-28-oic acid (DHUA), taraxerol, Ssqualene, sesquiterpenes and loliolide have been isolated in *T. catappa* [42,81,82].

### 5.6. Lignan Glucoside

Lignan glucosides are lignan compounds that are glycosylated (i.e., bound to sugar molecules, typically glucose). They are a class of polyphenolic compounds found in plants, particularly in seeds, fruits and bark steam, and are known for their antioxidant, anti-inflammatory and estrogenic properties [83]. Although *T. catappa* is known to contain various bioactive compounds, only a few studies reported the presence of specific lignans in this plant. Sowmya and Raveesha [84] reported the (8R, 8R) Secoisorlariciresinol 9-glucoside as a specific lignan glucoside in the leaves of *T. catappa*. Moreover, lignan glucosides are identified in other members of the *Terminalia* genus such as *T. chebula var.*, which showed the presence of termitomenins F and G and *T. citrina* containing *tomentella* and terminalosides [85,86].

### 5.7. Coumarins

Coumarins are a group of aromatic compounds with divers’ structure, found in various plants. These compounds are known for their potential therapeutic properties, including anti-inflammatory and anticoagulant effects [87]. Limited studies have reported coumarins in *T. catappa*. Those few studies include Mwangi et al. [41], who reported coumarins in *T. catappa* with demonstrated antimicrobial and antioxidant properties.

### 5.8. Steroids

**Figure 4:**
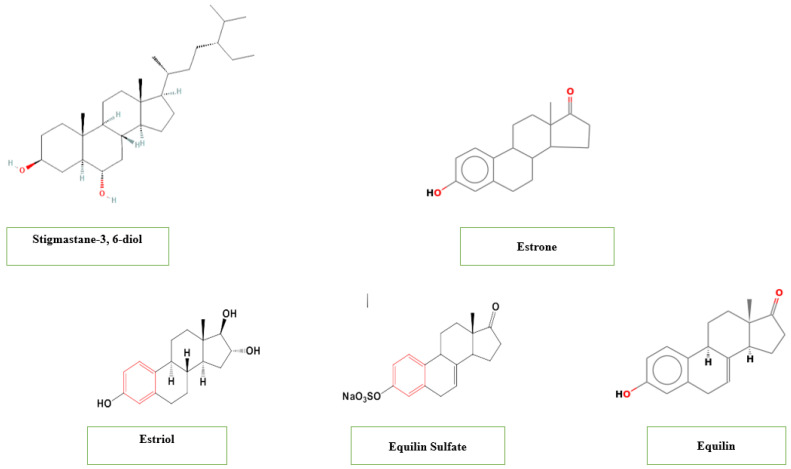
Steroids contained in *T. catappa*.

Steroids are organic compounds with a characteristic structure of four carbon rings called the steroid nucleus and play vital roles in biological processes in both plants and animals [88]. Several studies reported the presence of steroids in *T. catappa* at a concentration up to 8.07–6.24 3 mg/g reported in the fruits [41,80,89]. Such steroidal compounds include stigmastane-3, 6-diol identified in the stem barks and demonstrating antibacterial, antifungal, cytotoxic and antioxidant activities [82], and estrogenic steroids including estrone, estriol, equilin and equilin Sulfate (Figure 4) [82,90].

### 5.9. Polysaccharides (Galloyl Glucose and Derivatives)

Galloyl glucose and derivatives are a type of hydrolyzable tannin formed by the esterification of gallic acid with glucose, reported to be present in some spices of the Terminalia genus, including *T. myriocarpa*, *T. bellirica*, *T. chebula and T. arjuna*. [91] The galloyl glucose and derivatives reported in *T. catappa* include 2,6-digalloylglucose, 1,3,6-trigalloyl glucose, gallicacid3-O-6 galloyl glucoside and 1-*O*-galloylfructose. [84] These compounds were reported to exhibit antimicrobial activity with the potential to inhibit multidrug-resistant bacteria such as *Staphylococcus aureus*, and fungi such as *Trichophyton rubrum* and *Candida* spp., which are commonly detected in fermented foods. [84,92]

## 6. Bioactivities

### 6.1. Antioxidant Activity

The seeds, leaves, fruits, stems and bark of *T. catappa* are used for medicinal purposes, offering biological properties and high levels of polyphenolic compounds [93,94] (Table 3). Among the ethanolic extract from *T. Catappa* ripped fruit and leaves, the latter exhibited the highest levels of DPPH radical scavenging activity with an IC50 value of 43.34 µg/mL and reducing power potential, 2512.89 mM Fe (II)/g, respectively [95]. The significantly higher antioxidant properties in almond leaves, attributed to their rich content of flavonoids and tannins, suggest their more significant potential for managing oxidative stress and neurodegenerative conditions [94]. The antioxidant activity of *T. catappa* fruit flour (TCF) was measured as 13.06 μmol TEAC/g and 2.07 μmol TEAC/g using the ABTS and the FRAP method, respectively [6]. TCF, a source of polyphenolic compounds such as hesperidin, demonstrated activity against DPPH radicals [96]. Overall, antioxidant activity is associated with phenolic compounds such as rutin, quercetin and kaempferol [97,98,99]. Various studies on the antioxidant activity of oils have shown a strong linear correlation between phenolic content and antioxidant activity. Therefore, the high TPC values of *T. catappa* kernel oil (TCKO) from the Kota Kinabalu cultivar indicate its strong antioxidant activity [14]. Despite exhibiting comparable antioxidant properties, *T. catappa* remains an untapped and underutilized tree [2]. The GC-MS profiles of raw and roasted nuts revealed a promising abundance of naringin and flavan-3-ol, both known for their antioxidant potential [8]. Research suggests that polyphenols are the most abundant dietary antioxidants, contributing up to 90% of the total antioxidant capacity in most fruits and vegetables [100]. Numerous pharmacological studies have confirmed the antidiabetic and antioxidant activities of *T. catappa*, supporting its traditional uses [21,100]. *T. catappa* L. kernel oil from the purple oil variety exhibited notable antioxidant activity (38.6 ± 2.2 μg TE/g), as determined by the TEAC assay [3]. The ethanolic leaf extract of *T. catappa* exhibits anti-tumor activity with high antioxidant levels, likely attributed to its phenolic and flavonoid components [45]. Chakkalakal et al. [101] stated that tannin components from Indian almonds possess multiple antioxidant effects, capable of preventing lipid peroxidation (LPO) and superoxide formation. The methanol leaf extract of *T. catappa* was used to synthesize ZnO-NPs [73]. Both the extract and ZnO-NPs exhibited antioxidant scavenging activity, with the *T. catappa* extract showing better DPPH radical scavenging properties than the ZnO-NPs.

### 6.2. Antidiabetic Effect

Diabetes mellitus is a major global cause of mortality linked to hyperglycemia-induced blood disorders and thromboembolic risks [102]. *T. catappa* has a traditional use in both food and medicine [9]. All parts of *Terminalia catappa* are used in traditional medicine [103]. The seed kernel of *T. catappa* is nutrient-rich and can serve as a raw material for developing foods for diabetic patients [35]. Flavonoids and tannins in *T. catappa* seeds may enhance their anti-diabetic effects by (1) acting as antioxidants to reduce cellular damage [104], (2) improving insulin action, and (3) modulating glucose metabolism pathways [105]. Iheagwam et al. [106] investigated the effect of the aqueous extract of *T. catappa* leaf on hematological and coagulation disturbances in type 2 diabetic rats. The findings suggest it may reverse diabetes-related blood anomalies via anticoagulant and anti-anemic effects. *T. catappa* leaf aqueous extract suggests activating the Nrf-2 gene, an antioxidant-related gene [107]. As a result, hyperglycemia-induced oxidative stress and inflammation were reduced. Chinaka et al. [108] suggest that cis-9-hexadecanoic acid in *T. catappa* may enhance insulin sensitivity and prevent the destruction of insulin-secreting pancreatic beta cells. The extract powder of Vietnamese *T. catappa* leaves effectively inhibits α-glucosidase activity, making it a potential natural anti-diabetic agent [100]. GC-MS and FT-IR analysis of the n-hexane fraction identified compounds like eugenol, urs-12-en-24-oic acid, and squalene as potential antidiabetic agents, suggesting that *T. catappa* leaf extract contains α-glucosidase inhibitors and is a valuable source for natural anti-diabetic agents [109]. Despite its anti-diabetic and anti-inflammatory benefits, the aqueous extract of *Terminalia catappa* leaves may negatively affect male reproductive functions, altering sperm indices and hormones [110]. Because of its established therapeutic potential, it should be used with caution.

### 6.3. Anti-Inflammatory Effect

Rheumatoid arthritis, a chronic inflammatory autoimmune disorder, is characterized by ongoing synovial inflammation, bone and cartilage degradation, and joint destruction [111]. One of the factors contributing to rheumatoid arthritis is the protein. In vivo protein denaturation may be the cause of auto-antigen production in several rheumatic disorders. Electrostatic, hydrogen, hydrophobic and disulfide bonding changes are most likely part of the denaturation mechanism. Kumar et al. [112] in their study investigated the anti-inflammatory activity of *T. catappa* leaves aqueous extract. Data showed that the aqueous extract from *T. catappa* leaves can regulate the synthesis of auto-antigens caused by the in vivo denaturation of proteins in rheumatic disorders. Results thus support the importance of *T. catappa* leaves in the prevention and treatment of inflammatory disorders such as arthritis [112]. Likewise, protease inhibitory activity is another mechanism that the aqueous extract of *T. catappa* leaves exhibits anti-inflammatory properties. Synovial fibroblasts release proteases as the pannus spreads into adjacent bone and cartilage. These enzymes actively break down the collagen and proteoglycan matrix, contributing to the degradation of bone and cartilage [113]. Kumar et al. [112] demonstrated that the aqueous extract of *T. catappa* leaves exhibits significant anti-proteinase activity. Data showed that the aqueous extract of *T. catappa* leaves inhibited trypsin activity with IC_50_ values of 384.02 μg/mL. Human trypsin is activated in some types of rheumatoid arthritis, according to previous research [113].

### 6.4. Hepatoprotective Effect

The hepatoprotective properties of the *T. catappa* from Gwadar were examined and the results showed that the fruit part had hepatoproective effect [94]. In another study [94] investigating the hepatoprotective effect of *T. catappa*, the ethanol extract from the leaves helps mice whose livers have been acutely damaged by carbon tetrachloride (CCl4). Mice that received oral doses of 10 or 30 mg/kg before CCl4 injection showed a protective effect. The restoration of the altered activities of serum aspartate aminotransferase and sALT, which are markers of liver injury, showed a protective impact. Additionally, the study examined how the liver tissues of mice exposed to CCl4-induced hepatotoxicity changed in terms of the amount of IL-6 that appeared. The study’s conclusions suggest that the leaf extract’s protective effect against CCl4-induced acute liver damage in mice may be related to its ability to inhibit IL-6 overexpression in liver tissue [94].

### 6.5. Antimicrobial Activity

*T. catappa* has been shown to possess a range of pharmacological properties, including antibacterial properties [94]. Kumar et al. [112] investigated the antibacterial potential of *T. catappa* leaves aqueous extract using the agar well diffusion method against Gram-negative and Gram-positive bacteria. Generally, the extract exhibited a wide range of antimicrobial activity against every bacterium examined in this study, with notable differences noted between Gram-positive bacteria (*Staphylococcus* and *Bacillus* species), which are known to be more sensitive than Gram-negative bacteria (*E. coli*, *Klebsiella* species and *Pseudomonas* species). The greatest inhibitory impact was for Staphylococcus species in the Gram-positive group, and for *E. coli* in the Gram-negative group, with the zone of inhibition 12.0 mm and 9.5, respectively. However, among the bacteria that were studied, *Pseudomonas* spp. was shown to be the most resistant (5.5 mm) [112]. Other studies also showed that leaves and fruit extract have antibacterial properties against *Salmonella*, *Shigella*, *Enterococci* and *Corynebacteria* [94].

### 6.6. Anti-Atherosclerosis and Anti-Hyperlipidemia

Atherosclerosis is a condition where lipids build up in the arteries. Angina pectoris, myocardial infarction and stroke are the main side effects of atherosclerosis [114], whereas hyperlipidemia represents the early development of atherosclerosis and its cardiovascular complications [115]. The fruit of *T. catappa* demonstrated anti-atherosclerotic, anti-hyperlipidemic and hypolipidemic properties in the in vivo atherosclerosis model on Wistar rats [80]. According to the phytochemical screening, the ethanol and aqueous extracts of *T. catappa* fruit showed the presence of alkaloids, flavonoids, tannins, saponins, cardiac glycosides, phenols, terpenoids, anthraquinones and steroids. Tannins found in *T. catappa* act as anti-inflammatory agents, lowering the risk of atherosclerosis [80,116]. The findings of Tabansi et al. [80] demonstrated that in comparison to the healthy control group, which included treatment with *T. catappa* fruit extracts, the activity of alanine transaminase (ALT), aspartate transaminase (AST) and creatine kinase (CK) was considerably elevated under atherogenic settings. Data indicated that atherosclerosis had a regression impact after six weeks of *T. catappa* treatment. As well, *T. catappa* aqueous extract significantly decreased the activity of lactate dehydrogenase (LDH) when compared to atherogenic control. Likewise, the same study demonstrated that *T. catappa* fruit considerably reduced low-density lipoprotein (LDL) (200 and 300 mg/kg), indicating its effectiveness in blocking atherogenic pathways.

The antihyperlipidemic activity of the *T. catappa* fruit could be attributed to the significant amount of saponins in the extracts [80]. Saponins have antihyperlipidemic effects through their ability to interact with cholesterol in the intestinal lumen and reduce its absorption. The conversion of cholesterol to sterols by fecal secretion is accelerated by decreased bile acids in their extrahepatic circulation [117]. The in vivo treatment of *T. catappa* extract resulted in better histopathology, lipid profile, enzyme biomarkers, cardioprotective and atherogenic index [80].

### 6.7. Antitumor

The anti-tumorogenic activity of *T. catappa* is due to the free radical quenching property of its phytoconstituents [2]. *T. catappa* exhibited an antitumor effect by modulating lipid peroxidation and augmenting antioxidant defense systems in Ehrlich ascites carcinoma-bearing mice [45]. In experimental oncology, Ehrlich’s tumor is typically employed to assess the antitumoral activity of various natural compounds or for insight into the therapeutic potential of various synthesized chemotherapeutic drugs [118]. According to the result, the phenolic and flavonoid components in this extract may be responsible for antitumor activity [45].

### 6.8. Anthelmintic Activity

Paramphistomes are the cause of paramphistomosis, a serious helminthic disease that affects ruminants’ reticulums and rumens. Due to severe acute gastroenteritis, ulcerative ruminitis, dehydration, poor digestion, decreased nutrition conversion and decreased milk and meat production, the disease results in significant economic losses and a high host mortality rate, especially in young animals [119].

Minsakorn et al. [119] investigated the anthelmintic effect and bioassay-guided fractionation of *T. catappa* plant extracts against *G. Crumenifer* parasite. The results showed that the adult *G. crumenifer* could be suppressed by *T. catappa* leaf water extract (12–90% mortality); however, after 24 h of incubation, *T. catappa* leaf n-butanal extract demonstrated 100% mortality at 1000 μg/mL. As well, the anthelmintic activity of the *T. catappa* plant has been reported recently in a published study [94].

### 6.9. Antimalarial Activity

Malaria is one of the most deadly parasitic infectious diseases, which is caused by *Plasmodium* protozoa, and in many tropical places, notably West African nations, it is one of the main causes of mortality [120]. In 2023, there were an anticipated 263 million cases and 597,000 malaria deaths globally, according to the World Health Organization’s most recent World Malaria Report [121].

Meanwhile, specific recent studies directly assessing the antimalarial activity of *T. catappa* were not identified. The plant is well-known for having a high concentration of flavonoids and phenolic chemicals, [2] which have been linked to antimalarial qualities in other plant species. For instance, *Terminalia mantaly* H. Perrier belongs to the same family of *T. catappa*, which is Combretaceae, [2,120], and has been reported to have antimalarial properties against in vitro β-hematin synthesis. Therefore, it is suggested that *T. catappa* could have antimalarial activities, and further research is needed to explore this potential.

In short, the bioactivity of *T. catappa* is largely attributed to its high concentration of bioactive compounds, which explains its potent antioxidant, antimalarial, antimicrobial, antidiabetic, hepatoprotective, anti-atherosclerosis and anti-inflammatory properties. The phytochemical and bioactivity properties richness make *T. catappa* a promising source for functional foods, nutraceuticals, pharmaceuticals and cosmetic studies.

**Table 3:** Biological properties of different parts of *T. catappa*.

Biological Activity	Plant Organs	Main Findings	Reference(s)
**Antioxidant activity**	Seeds	-The low peroxide value of the oil confirmed the presence of antioxidants in the seed oil. -*T. catappa* kernel flour showed significant antioxidant properties.	[105,122]
	Nuts	Adding almond nut flour to kunu beverage improved its antioxidant properties.	[123]
**Antidiabetic activity**	Leaf	High antidiabetic activities were related to higher phenolic constituents of almond leaf.	[124]
	Seed	-Modulation of lipid profile. -LDL reduction.	[125]
	Fruit	Methanolic and aqueous extracts exhibited significant antihyperglycemic activities.	[126]
**Anti-atherosclerotic, Anti-hyperlipidemic**	Fruit	-Tannins found in *T. catappa* act as anti-inflammatory agents, lowering the risk of atherosclerosis. -Reduced low-density lipoprotein (LDL) (200 and 300 mg/kg).	[80]
**Antitumor activity**	Leaves	*T. catappa* exhibited antitumor effect by modulating lipid peroxidation and augmenting antioxidant defense systems in Ehrlich ascites carcinoma bearing mice.	[2,45]
**Anthelmintic activity**	Leaves	-*G. crumenifer* parasite could be suppressed by *T. catappa* leaf water extract (12–90% mortality).	[94]
**Anti-inflammatory activity**	Leaves	-*T. catappa* leaves can regulate the synthesis of auto-antigens caused by the in vivo denaturation of proteins in rheumatic disorders. -Showed protease inhibitory activity.	[112]
	Leaves and fruit	-Antibacterial properties against *Staphylococcus*, *Bacillus* spp. *E. coli*, *Klebsiella* spps, *Pseudomonas salmonella*, *shigella*, *enterococci* and *corynebacteria*.	[94,112]
**Hepatoprotective effect**	Leaves and fruit	*T. Catappa*, the ethanol extract from the leaves helps mice whose livers have been acutely damaged by carbon tetrachloride (CCl4).	[94]
**Antimicrobial activity**	Leaves	-Gram-positive bacteria (*Staphylococcus* and *Bacillus* species) are more sensitive than Gram-negative bacteria (*E. coli*, *Klebsiella* species and *Pseudomonas* species).	[94,112]

## 7. Economic Potentials

The global market for products derived from *T. catappa* is growing significantly, with important economic implications at various levels. The global market for products derived from *T. catappa* was valued at 450 million USD in 2023, with growth projected to 750 million USD by 2028 [82]. As estimated global market value, the market segments break down as follows: (i) Pharmaceuticals: 45% (202.5 million USD), (ii) Cosmetics: 30% (135 million USD), (iii) Food products: 15% (67.5 million USD) and (iv) Environmental applications: 10% (45 million USD). Trade in *T. catappa* and its derivatives show positive trends, with global exports of up to 280 million USD (2023). The main exporting countries are India (35%), Indonesia (25%) and the Philippines (15%), with countries such as the United States (30%), the European Union (25%) and Japan (20%). *T. catappa* harvesting generates significant income for local communities, with an average income estimated at between 1200 and 2500 USD/year per grower. Around 50,000 direct jobs have been created in producing countries, and the activity contributes between 0.5 and 1.2% to the agricultural GDP of the main producing regions. In Indonesia, for example, processing cooperatives saw their revenues increase by 35% between 2020 and 2023 thanks to the valorization of by-products [9].

## 8. Challenges and Prospects

### 8.1. Current Challenges

Standardization and quality control are major obstacles in the exploitation of *T. catappa*. Recent studies have highlighted significant variations in the concentration of bioactive compounds, with fluctuations of up to 25–40% depending on geographical region and harvesting conditions. This variability poses considerable challenges for the standardization of extracts and their use in commercial formulations. The lack of standardized extraction protocols also represents a major constraint, with efficiency rates varying considerably between 45 and 85% depending on the methods used [127].

In economic terms, the current market structure presents a number of limitations. Production costs absorb a significant proportion of revenues, representing between 30 and 40% of total sales. Access to international markets remains restricted for many producers, particularly in developing countries. Price volatility, characterized by variations of up to 25% over twelve months, creates additional uncertainty for industry players [9].

Environmental issues also represent a major challenge. Growing pressure on natural resources, exacerbated by increasing global demand, raises questions about the long-term sustainability of *T. catappa* farming. The impacts of climate change on production are beginning to show, affecting growth cycles and the quality of bioactive compounds. The need to adopt more sustainable practices is therefore becoming imperative to ensure the sustainability of the resource.

### 8.2. Prospects

Recent technological advances open up promising prospects for the development of new pharmaceutical formulations. Ongoing research into innovative extraction methods, notably the use of ultrasound and microwaves, holds out the prospect of significant improvements in extract yields and quality. Developments in biotechnology, particularly in the field of personalized medicine, offer new opportunities for exploiting the therapeutic properties of *T. catappa* [128]. Commercial expansion is a major development focus. Current research into by-product valorization is paving the way for new industrial applications. The development of high-value-added products, combined with certification and labeling strategies, could lead to greater penetration of international markets. This diversification of commercial applications is accompanied by innovations in processing and packaging, aimed at improving the stability and bioavailability of active compounds.

The socio-economic dimension also offers encouraging prospects. The development of the *T. catappa* sector represents a significant opportunity for job creation along the entire value chain. The emergence of local entrepreneurial models, supported by training programs and technical support, could help to structure the sector. The expected increase in exports, combined with a better valuation of products on international markets, suggests a positive impact on local economies.

**Figure 5:**
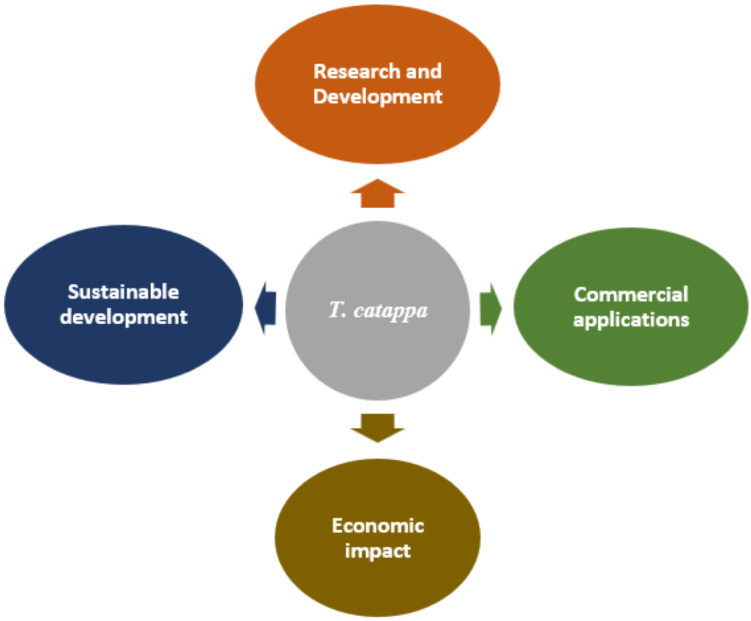
Integrated framework for the development of *T. catappa*.

Environmental sustainability is becoming a key strategic focus. Initiatives for the conservation of genetic resources and the implementation of sustainable agricultural practices bear witness to a growing awareness of environmental issues. The development of more environmentally friendly production methods, including the optimization of extraction processes and the reduction of carbon footprints, is becoming a priority to ensure the long-term viability of the sector. An integrated framework for the sustainable development of *T. catappa* should be explored (Figure 5).

## 9. Conclusions

An in-depth analysis of *T. catappa* reveals its significant potential as a valuable natural resource with diverse applications. Scientific evidence confirms its traditional uses while identifying new development opportunities. The different parts of T. catappa are rich sources of nutrients and bioactive compounds. Furthermore, they displayed tremendous biological activities, including antioxidant, antidiabetic, anti-atherosclerosis, antitumor, antimicrobial, anthelmintic, antimalaria, hepatoprotective, insecticidal, anti-inflammatory and antihyperlipidemic activities. Integrating sustainable practices with modern scientific approaches offers a promising route to maximizing the benefits of this versatile species. However, success in realizing these opportunities will require continued investment in research, improved standardization processes and effective collaboration between stakeholders. Future research directions should focus on resolving current challenges while exploring innovative applications that can contribute to both economic development and environmental sustainability.

## Data Availability

Data sharing does not apply to this article as no new data were created or analyzed in this study.
